# Hepatitis C Virus Deletion Mutants Are Found in Individuals Chronically Infected with Genotype 1 Hepatitis C Virus in Association with Age, High Viral Load and Liver Inflammatory Activity

**DOI:** 10.1371/journal.pone.0138546

**Published:** 2015-09-25

**Authors:** Cristina Cheroni, Lorena Donnici, Alessio Aghemo, Francesca Balistreri, Annalisa Bianco, Valeria Zanoni, Massimiliano Pagani, Roberta Soffredini, Roberta D’Ambrosio, Maria Grazia Rumi, Massimo Colombo, Sergio Abrignani, Petra Neddermann, Raffaele De Francesco

**Affiliations:** 1 INGM, Istituto Nazionale Genetica Molecolare “Romeo ed Enrica Invernizzi”, Milan, Italy; 2 A.M. Migliavacca Center for Liver Disease, First Division of Gastroenterology, Fondazione IRCCS Ca’ Granda Ospedale Maggiore Policlinico Milano, Università degli Studi di Milano, Milan, Italy; 3 Division of Hepatology, Ospedale San Giuseppe IRCCS Multimedica, Università degli Studi di Milano, Milan, Italy; 4 DISCCO, Department of Clinical Sciences and Community Health Università degli Studi di Milano, Milan, Italy; University of Padua, ITALY

## Abstract

Hepatitis C virus (HCV) variants characterized by genomic deletions in the structural protein region have been sporadically detected in liver and serum of hepatitis C patients. These defective genomes are capable of autonomous RNA replication and are packaged into infectious viral particles in cells co-infected with the wild-type virus. The prevalence of such forms in the chronically HCV-infected population and the impact on the severity of liver disease or treatment outcome are currently unknown. In order to determine the prevalence of HCV defective variants and to study their association with clinical characteristics, a screening campaign was performed on pre-therapy serum samples from a well-characterized cohort of previously untreated genotype 1 HCV-infected patients who received treatment with PEG-IFNα and RBV. 132 subjects were successfully analyzed for the presence of defective species exploiting a long-distance nested PCR assay. HCV forms with deletions predominantly affecting E1, E2 and p7 proteins were found in a surprising high fraction of the subjects (25/132, 19%). Their presence was associated with patient older age, higher viral load and increased necroinflammatory activity in the liver. While the presence of circulating HCV carrying deletions in the E1-p7 region did not appear to significantly influence sustained virological response rates to PEG-IFNα/RBV, our study indicates that the presence of these subgenomic HCV mutants could be associated with virological relapse in patients who did not have detectable viremia at the end of the treatment.

## Introduction

Infection with the hepatitis C virus (HCV) is believed to affect about 2% of the world population and represents the major cause of chronic liver disease, liver cirrhosis and hepatocellular carcinoma [1]. HCV is an enveloped virus belonging to the family of *Flaviviridae*. Its genome consists of a positive-stranded RNA molecule of approximately 9.6 Kb that is structured in a 5’ untranslated region (UTR), a long open reading frame encoding a polyprotein precursor of about 3010 amino acids and a 3’UTR region. The polyprotein is processed to produce structural (Core and E1-E2 glycoproteins) and non-structural (p7, NS2, NS3, NS4A, NS4B, NS5A and NS5B) proteins. A characteristic of HCV is its remarkable genetic diversity, resulting in the diversification of virus genome into six major genotypes as well as the typical presence of quasispecies in infected patients. The RNA-dependent RNA polymerase NS5B is a low fidelity enzyme; this characteristic, coupled with the lack of proofreading ability and the extremely high virion turn over rate, is believed to be the driving force of HCV genetic diversity [2].

HCV variants characterized by large genomic deletions have been episodically reported to be detected in sera or liver biopsy specimens of patients with chronic hepatitis C [3–8]. These circulating HCV subgenomes typically contain a deletion variably affecting the E1-NS2 region, but retain the Core region in addition to the genome portions that are essential for autonomous HCV replication (5’-UTR, NS3-NS5B and 3’-UTR). Importantly, such genomic deletions are invariably found to maintain the original open reading frame (ORF) and therefore the ability to produce a polyprotein. Using an *in vitro* infectivity model, it was demonstrated that these subgenomic HCV RNAs are capable of autonomous replication and are packaged into infectious viral particles when co-expressed together with full-length viral genome [7,9]. Thus, the subgenomic HCV species share biological and structural properties with the defective interfering (DI) viruses reported for other RNA viruses, including the *Flaviviridae* family members pestivirus (Classical Swine Fever Virus, CSFV, and Bovine Viral Diarrhoea Virus, BVDV [10,11]) and flavivirus (Murray Valley encephalitis virus [12]).

Although one study reported that putative DI HCV were found circulating in a significant fraction of patients infected with different viral genotypes [6], the prevalence of these HCV deletion mutants has never been systematically and carefully evaluated in well-characterized cohort(s) of HCV-infected subjects. In addition, while a key pathogenic role of DI forms is well-established for CSFV and BVDV, the clinical relevance of HCV particles with deleted genomes remains to be assessed. Notably, it has been suggested that the presence of DI HCV clones could be related to disease severity [7] or progression [3] in chronic hepatitis C, and that the emergence of HCV with large sub-genomic deletions could be associated with failure to respond to PEG-IFNα/RBV therapy in patients with recurrent hepatitis C after liver transplantation [4]. Moreover, a recent study performed by next generation sequencing on a small group of patients undergoing liver transplant recipients revealed the presence of this kind of HCV deletion mutants in 4 of 5 patients, suggesting they might occur with high frequency in HCV patients with end-stage liver disease [8]. All these suggestions, however, remain speculative in nature, since they are based on sporadic observations made in very small number of patients and in the absence of extensive clinical data.

In order to assess the prevalence at which circulating HCV particles with deleted genomes occur in a chronically HCV-infected population and how they relate to severity of liver disease as well as other virologic or clinical characteristics, we initiated a screening campaign aimed at the detection of HCV defective genomes in serum samples obtained from well-characterized cohorts of chronic hepatitis patients. In this study, we report the clinical characteristics that appear to be associated with the presence of circulating HCV deletion mutants in patients chronically infected with genotype 1 HCV (HCV1). Our study reveals a potential correlation between the presence of the defective-interfering viral genomes and patient age, viral load, the presence of liver necroinflammatory lesions and the pattern of treatment failure to PEG-IFNα/RBV treatment.

## Materials and Methods

### Patients

152 patients infected by genotype 1 HCV who received PEG-IFNα-2b plus RBV therapy in a previous study [13] conducted at the Center for Liver Disease at Maggiore Hospital (Milan, Italy) were offered participation in the current study. Treatment exclusion criteria were Hepatitis B Virus or Human Immunodeficiency Virus co-infection, decompensated liver disease, drug dependence or >40 g/day alcohol intake. Subjects affected by poorly controlled diabetes, severe depression, autoimmune disease or concomitant malignant neoplastic diseases were also excluded. A diagnostic liver biopsy performed prior to antiviral treatment was available for each subject. Liver biopsies were evaluated by a single expert pathologist and scored according to the Ishak system in separate reports for grading (from 0 to 18, ranging from 0 to 4 for piecemeal necrosis, focal necrosis and portal inflammation, and from 0 to 6 for confluent necrosis) and staging (from 0-no fibrosis- to 6-cirrhosis-).

Serum HCV RNA was assessed by in-house nested reverse transcriptase (RT)-PCR, using specific primers for 5’UTR and quantified by Versant HCV RNA 3.0 assays (bDNA 3.0; Bayer Corporation, Emeryville, CA, USA). Excluded from the current analysis were 12 patients who discontinued anti-HCV treatment prematurely for non-virological reasons, as well as 8 patients who were lost to follow-up. A total of 132 HCV1 were enrolled and analyzed.

The study protocol conformed to the Declaration of Helsinki and was approved by the Institutional Review Board of the Fondazione IRCCS Ca' Granda. Each patient signed a written informed consent.

The demographic and selected clinical traits of the patient population are described in Table 1.

**Table 1:** Demographic and clinical features of the 132 HCV1 patients, stratified by presence of HCV defective particles or IL28B genotype.

Factor	All patients (n = 132)	Stratified by HCV defective presence			Stratified by IL28B genotype		
		Full-length (n = 107)	Defective (n = 25)	Pval	rs12979860 CC (n = 56)	rs12979860CT+TT (n = 76)	Pval
Age (years)	56 ± 19.25	54 ± 22	57 ± 10	**0.0367**	55 ±19.75	56 ±19	NS
HCV RNA (IU/ml)	740820.5± 1004706	703751 ± 859449	1350000 ± 1443900	**0.0046**	700032.5± 858765	778654 ± 1004706	NS
ALT (IU/l)	103 ± 97.5	101 ± 95	108 ± 126	NS	123 ± 155	91.5 ± 65.5	**0.0133**
GGT (IU/l)	60 ± 63.25	60 ± 62	46 ± 76	NS	45 ± 60	72.5 ± 81	**0.0316**
Male gender	69 (52.3%)	57 (53.3%)	12 (48.0%)	NS	27 (48.2%)	42 (55.3%)	NS
BMI ≥ 25 kg/m^2^	43 (32.6%)	36 (33.6%)	7 (28.0%)	NS	17 (30.4%)	26 (34.2%)	NS
Grading ≥ 9	40 (30.3%)	26 (24.3%)	14 (56.0%)	**0.003**	22 (39.3%)	18 (23.7%)	NS
Staging ≥ 4	49 (37.1%)	38 (35.5%)	11 (44.0%)	NS	19 (33.9%)	30 (39.5%)	NS
Cirrhosis	35 (26.5%)	29 (27.1%)	6 (24.0%)	NS	13 (23.2%)	22 (28.9%)	NS
IL28B CT/TT	76 (57.6%)	66 (61.7%)	10 (40.0%)	NS	—	—	—

Continuous variables are expressed as median ± interquartile range (IQR). P values are calculated by Wilcoxon test and Fisher's test for continuous and categorised variables respectively; NS: not significant.

### Treatment

Patients were treated for 48 weeks with PEG-IFN-α2b (PEGINTRON) at doses of 1.5 μg/kg once per week subcutaneously. RBV (REBETOL) was dosed according to baseline weight (800 mg for weight <65 kg, 1000 mg for 65–85 kg and 1200 mg for >85 kg). Clearance of serum HCV RNA was assessed at week 4 (RVR: rapid virological response), at week 12 (cEVR: complete early virological response) and at week 48 of treatment (ETR: end of treatment response). Sustained virological response (SVR) was defined as undetectable HCV RNA at post-treatment week 24. ETR-positive patients who became HCV-RNA positive during the follow-up were classified as relapsers. Patients who had any other virological response were considered as non-responders.

### Viral RNA extraction and amplification

Screening for the presence of HCV defective species was performed exploiting long-distance nested PCR amplification [from nucleotide 127 (5’UTR) to 3649 (NS3), see below]. Degenerated primers specific for genotype 1 were manually designed on the consensus sequence of 1234 HCV genotype 1 complete sequences downloaded from HCV sequence database (http://hcv.lanl.gov/content/index).

Total RNA was extracted from 200 μl of serum (obtained prior to initiation of treatment) by High Pure Viral RNA kit (Roche Diagnostics GmbH). Reverse transcription was performed with SuperScript® III First-Strand Synthesis System (Life Technologies) using degenerate genotype 1 HCV-specific primer TGGTCYACATTGGTRTACATYTG (nt 3636–3658) according to manufacturer’s instructions. cDNA fragments were amplified by nested PCR with Platinum® Taq DNA Polymerase High Fidelity (Life Technologies). The first round of amplification was performed with 1 μl of template and primers sense ATCACTCCCCTGTGAGGAAC (nt 36–55) and antisense TGGTCYACATTGGTRTACATYTG (nt 3636–3658) in 25 μl of reaction volume. For the second round of amplification, 1 μl of template and primers sense TCCCGCGAGAGCCATAGT (nt 127–144) and antisense TTGGTRTACATYTGGRTGAYHGG (nt 3627–3649) were used. PCR cycling conditions consisted of a step of 94°C for 2 min followed by 35 cycles of 15 sec at 94°C, 90 sec at annealing temperature (from 48°C to 55°C), 4 min at 68°C and final elongation 10 min at 68°C. To improve the yield, the first 10 PCR cycles were performed at a lower annealing temperature and the following 25 cycles by improving the elongation time for 5 sec for every cycle. As positive control, part of Core sequence was amplified with AmpliTaq Gold DNA Polymerase (Life Technologies) using primers sense AGGTCTCGTAGACCGTGCATCATG (nt 321–344) and antisense CAYGTRAGGGTATCGATGAC (nt 705–724).

PCR products were examined on 1% agarose gel stained with ethidium bromide. Amplicons showing higher mobility compared to HCV full-length genome were gel purified with Wizard SV Gel and PCR Clean-Up System (Promega), and subjected to DNA sequence analysis. Sequences were analyzed by BioEdit and Vector NTI software. HCV genome of H77 strain (accession NC_004102) was used as reference sequence.

### Titration of synthetic full length/defective HCV RNA mixtures

Synthetic HCV RNA was prepared from pCR-Blunt II- TOPO cloned samples with defective or full length species from a serum sample (#131) following MEGAscript® T7 Transcription Kit (Life Technologies) manufacturer's procedures. Obtained RNA was subjected to qualitative control and quantitative determination by fluorimetric and spectrophotometric techniques. Different full length-defective RNA mixtures, with variable ratios of the two components, were prepared by keeping the total maximum number of RNA copies constant at 500.000 UI/ml, corresponding to an intermediate level of viremia value. Mixture samples were thus prepared with the following full length-defective ratio values: 1, 2.5, 5, 7.5, 10, 25, 50, 75, 100, 1000; control samples containing only full length or defective species were also used. Synthetic mixtures were subjected to RT-nested PCR as previously described and loaded on agarose gel.

### Quantitative analysis of defective/full length HCV genome

The levels of total or defective HCV genome copies where quantitatively determined in six patients for which larger amounts of serum were available (patients 117, 124, 143, 159, 171 and 182). For these patients, amplicons corresponding to the full-length HCV RNA as well as amplicon corresponding to the deleted species were gel purified with Wizard SV Gel and PCR Clean-Up System (Promega), cloned into pCR-Blunt II- TOPO (Zero Blunt TOPO PCR Cloning Kit; Life Technologies) following manufacturer's instructions and subjected to DNA sequence analysis. The level of HCV RNA in the patients sera was determined by two-step real time PCR with TaqMan® Universal PCR Master Mix (Life Technologies). Reverse transcription was performed with SuperScript® III First-Strand Synthesis System (Life Technologies) as previously described. A 384-well-plate-based assay was performed with 2 μl of obtained cDNA in 10 μl of reaction per well. To quantify total HCV RNA a set of primers and probe annealing in 5'UTR common region was used, while to quantify defective HCV species different specific sets matching the junction region of each defective sample were designed. In particular the following primers and probe assay were used: HCV-GP1.GP2 (general set matching 5'UTR region that recognizes all constructs), sense (5-GCGAAAGGCCTTGTGGTACT-3), antisense (5-CACGGTCTACGAGACCTCCC-3), and probe (5[FAM]-CCTGATAGGGTGCTTGCGAGTGCC-3[TAMRA]); R117 (specific set of sample 117), sense (5-CTCCGCGTGCGAAGTG-3), antisense (5-CCGAGTATGGCGAGCAAGA-3), and probe (5[FAM]-CATGTCACCATCACCAAAC-3[NFQ]); R124 (specific set of sample 124), sense (5-GATCTGCCCTCCTCGTTTCC-3), antisense (5-CCATGAGTGGGCCGAGTATG-3), and probe (5[FAM]-CTGTTCACCAAACTCTTG-3[NFQ]); R159 (specific set of sample 159), sense (5-ACTGCAACTGCTCGATCTATCC-3), antisense (5-CCACCATATGATCTTAGCGAGGAC-3), and probe (5[FAM]-ACGTACCACACTATAAAAG-3[NFQ]); R143 (specific set of sample 143), sense (5-CGTACCACGTCACGAACGA-3), antisense (5-GCGAGCAAGATTTTGGTGAACAC-3), and probe (5[FAM]-AAGGCACGCGGAGTTG-3[NFQ]); R182 (specific set of sample 182), sense (5-CCGCCTATGAAGTGCACAAC-3), antisense (5-CATGAGCGGACCGAGTATGG-3), and probe (5[FAM]-CAAGAGGTTGGTGATGTACAC-3[NFQ]); R171 (specific set of sample 171), sense (5-CGGCTAGGAACACCAGTGT-3), antisense (5-GCAGCACAGAAGAACACAAGGA-3), and probe (5[FAM]-TACGCATGGCATCCTC-3[NFQ]).

All reactions were run in quadruplicate with 7900HT Fast Real-Time PCR System (Life Technologies) under the following conditions: 10 min at 95°C, 40 cycles of 15 s at 95°C and 1 min at 60°C. Quantitative calculations were obtained using the absolute standard curve method (described in Applied Biosystems 7900HT Fast Real-Time PCR System, absolute quantitation using standard curve guide, PN4364014).

Synthetic HCV RNA was prepared from each pCR-Blunt II- TOPO cloned sample with defective or full-length species following MEGAscript® T7 Transcription Kit (Life Technologies) manufacturer's procedures. Titration curve was prepared with serial 5-fold dilutions of synthetic HCV specific RNA followed by retrotranscription, in order to obtain a standard curve of cDNA spanning from 100 to 1.56×10^6^ HCV genome copies. Quantification of defective and total HCV RNA was expressed in copies per ml of serum.

### Genomic DNA extraction and determination of IL28B genotype

Genomic DNA was extracted from 100 μl of serum using QIAamp DNA Micro kit (Qiagen) and pre-amplified with Genoplex Whole-genome Amplification kit (Sigma) following manufacturer’s instructions. rs12979860 TaqMan SNP Genotyping assays were run starting from 30 ng of pre-amplified DNA on a 7900HT real time PCR instrument (Applied Biosystems, Carlsbad, CA), following manufacturer’s instructions.

### Statistical analysis

Continuous variables were expressed as median ± interquartile range (IQR), while categorized variables were expressed as frequencies; statistical comparisons between groups were performed by the Wilcoxon test (also known as Mann-Withney test) and Fisher’s test respectively. The threshold of significance was set at 0.05 for all the analyses.

Multivariable logistic regression was used in order to evaluate the effect that categorical or continuous explanatory variables have on a categorical binary outcome. Briefly, multivariable logistic regression models were generated for the study of high histological grading, ETR or relapse as outcomes. In order to select the variables that are more relevant for the outcome of interest (and therefore to include in the final logistic regression model), an automatic stepwise algorithm was employed. The stepwise algorithm inserts and deletes explanatory variables fed by the operator in an iterative process in order to identify a final model on the basis of the minimum Akaike Information Criterion [14] (AIC). AIC is used in model selection procedure as a measure of the quality of the generated model and it represents a trade-off between the goodness of fit of the model and its complexity. Variables considered as input for the stepwise selection process were as detailed below. For hystological grading ≥ 9 as outcome, we considered patient age, viral load, gender, BMI ≥ 25 Kg/m^2^, IL28B genotype, and the presence of HCV defective variants. For ETR as outcome, we considered patient age, viral load, gender, BMI ≥ 25 Kg/m^2^, IL28B genotype, presence of HCV defective variants, grading ≥ 9, staging ≥ 4 (or cirrhosis), ALT, and GGT. For virological relapse as outcome, we considered patient age, viral load, gender, BMI ≥ 25 Kg/m^2^, IL28B genotype, presence of HCV defective variants, grading ≥ 9, staging ≥ 4 (or cirrhosis), ALT, and GGT. The final models obtained by applying the described procedure for the three considered outcomes are reported in the result sections. All statistical analyses were performed in R [15], using MASS library for the stepwise procedure [16].

## Results

### Defective viral genomes are present in the serum of a large fraction of chronic HCV1 patients

Pre-therapy serum samples of 132 subjects selected based on the selection criteria were screened for the presence of HCV defective species. Demographic and clinical characteristics of the patients of this study are summarized in Table 1. Both genders were well represented (52.3% males) and the median age was 56 years (IQR 19.25). 30.3% of patients had moderate to severe histological activity (Grading ≥ 9) and 37.1% of them showed advanced fibrosis (Staging ≥ 4). RVR and cEVR were obtained respectively in 34.1% and 58.3% of the cohort. At the end of treatment and follow-up period, 47 subjects (35.6%) achieved SVR, while 27 experienced a viral relapse and 58 were non-responders.

**Fig 1:**
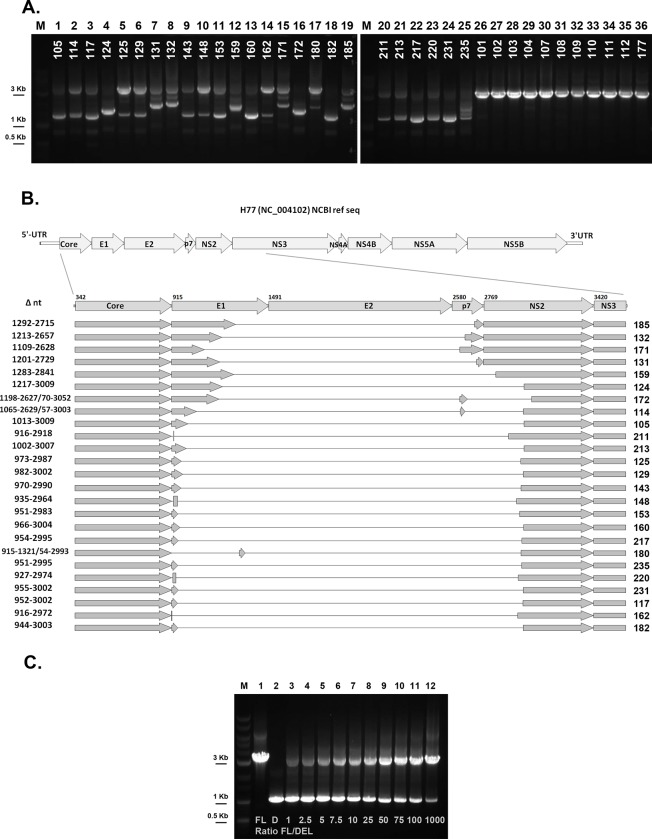
Viral genome architecture of the defective forms identified in the serum of chronic hepatitis C patients (genotype 1 HCV). (A) Pictures of agarose-gel showing amplicons obtained after the second round of nested PCR for all the patients in which defective forms were identified (lanes 1–25) and a subset of patients negative for the defective form (lanes 26–36). Lanes M: molecular size markers. (B) Schematic representation of the architecture of the 25 defective variants identified in the sera of subjects chronically infected with genotype 1 HCV. (C) Picture of agarose gel showing amplicons obtained after nested PCR performed on synthetic HCV RNA mixtures assessing full-length/defective ratios from 1 to 1000 (lanes 3–12). FL: full-length RNA only (lane 1); D: deleted RNA only (lane 2). Lane M: molecular size markers.

For all specimen used, the long-range nested PCR amplification of viral RNA produced one or more amplicons. For several subjects, an amplicon of a smaller size than expected from the amplification of full-length HCV genome could be observed (1200–2000 bps vs. expected size of 3522 bps, Fig 1A). This result suggested that, in these patients, a fraction of the circulating viral RNA contains a large genomic deletion located between the Core and the NS3 regions. The shorter-than-expected amplicons were then isolated and subjected to DNA sequencing. The presence of HCV genomes with large genomic deletions was confirmed by nucleotide sequencing in 18.9% of the patients (25/132). Importantly, all the genomic deletions that we identified in this study maintained the original ORF, indicating the potential to support translation from the authentic initiation codon. The results of the nucleotide sequence analysis are summarized in Fig 1B. In all of the defective variants, E2 was completely deleted while E1 and p7 were at least partially missing; the initial portion of NS2 was lost in 21/25 defective genomes (84%). Double deletions in a single genome were observed in 3 patients, in which a small residual fragment ranging from 28 to 43 bps was localized between the two large deletions (Fig 1B, samples 172, 114, 180).

In order to evaluate the sensitivity of our screening assay to the presence of defective genomes, the protocol was evaluated using synthetic HCV RNA mixtures assessing full-length/defective ratios ranging from 1 to 1000 (Fig 1C). As expected, the shorter, defective specie is amplified more efficiently and is detected as the most intense band for full-length/defective ratios ranging from 1 to 10. The amplicon corresponding to the deleted genome is still clearly detectable when it represents only the 0.1% of the total HCV RNA copies, thus suggesting that the non-quantitative PCR-based assay would detect as low as 0.1% of deleted HCV genome in a sample with intermediate viremia (see also Materials and Methods).

The results obtained by the nested PCR screening described above provide a qualitative result, indicating the presence of defective form with high sensitivity; however, due to the preferential amplification of the shorter form, it does not give information on the quantity of the full-length versus the defective form. To address this point, a subset of patients who carried defective form were subjected to quantitative detection of HCV genomes by real-time PCR. In each sample, the total HCV was measured using a primer-probe set matching the 5’UTR region of the viral genome, that is conserved across samples and maintained also in defective genomes. The defective form was estimated using a primer-probe set designed specifically for each patient on the unique junction region identified from sequencing of the clone. The results are summarized in Table 2.

**Table 2:** Quantitative detection of total and defective HCV RNA in a subset of patients who carried defective HCV forms.

Patient	Total HCV copies/ml	Defective HCV copies/ml	% Defective/Total
**171**	1.13E+06	4.80E+04	**4.3%**
**117**	1.14E+06	1.99E+05	**17.5%**
**124**	5.30E+04	9.98E+03	**18.8%**
**159**	2.25E+06	6.96E+05	**30.9%**
**143**	5.69E+05	3.24E+05	**56.9%**
**182**	7.03E+06	4.93E+06	**70.1%**

Briefly, the abundance of the defective form compared to total HCV is very heterogeneous in the subset of analyzed patients, spanning from about 4% to about 70%. In 2 out of 6 patients, the defective form is more than 50% of total HCV, indicating that deleted genomes may represent an important part of circulating HCV.

### The presence of HCV defective genomes is associated with patient older age, higher viral load and liver necroinflammatory activity

**Fig 2:**
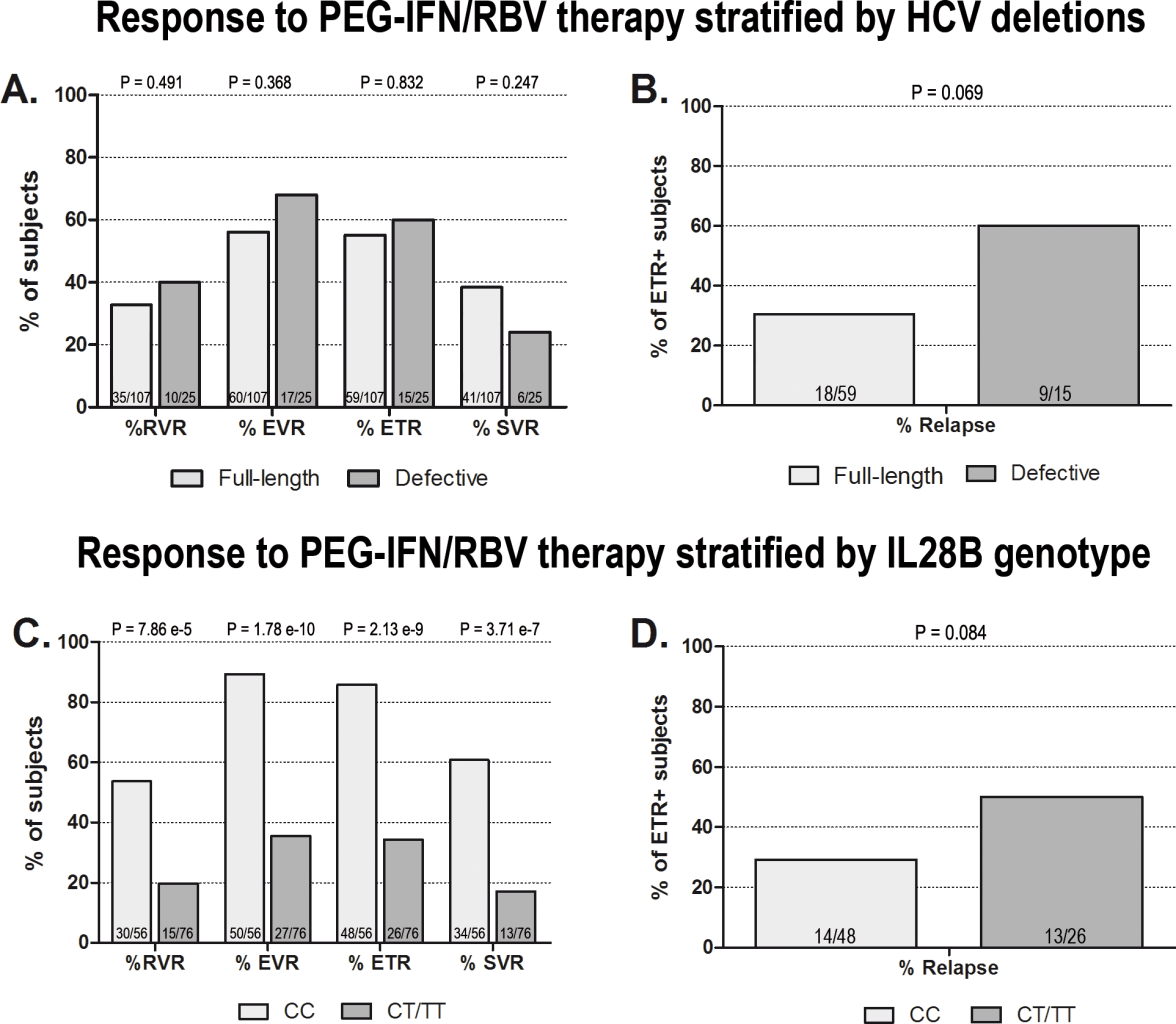
Virogical responses to PEG-IFNα/RBV stratified for HCV defective forms or IL28B genotype. RVR, EVR, ETR and SVR rates in the overall population as well as relapse rates in ETR-positive subjects, according to the presence of HCV deletions (A-B) or IL28B genotype (C-D), are reported. The presence of HCV defective particles does not have a significant effect on RVR, EVR, ETR or SVR rates, while it correlates with a higher probability of relapse in ETR-positive subjects (A-B). IL28B CT/TT genotypes are significantly associated with lower RVR, EVR, ETR and SVR rates and correlate with a higher probability of relapse (C-D).

We next examined the correlation between the presence of defective HCV forms and clinical characteristics in the study cohort. As illustrated in Table 1, by univariate analysis, the presence of HCV defective genomes was found to be significantly associated with patient older age (Wilcoxon test Pval 0.0367), higher viral load (Wilcoxon test Pval 0.0046) and with mild to severe hepatic necroinflammatory activity (histological activity index ≥ 9; Fisher’s test Pval 0.003). Analyzing viral kinetics during PEG-IFNα/RBV treatment, we could not detect substantial differences in the decline of viral load at early time-points (RVR and cEVR rates) in carriers of HCV deleted variants; also ETR percentages were unchanged. SVR rates were lower for patients with defective genomes (24% compared to 38.3%), but the difference was not found to be statistically significant (Fisher test Pval 0.247, Fig 2A). Notably, however, among 74 patients who were HCV RNA-negative at the end of the treatment, virological relapse was observed in 60% (9/15) of the subjects carrying HCV defective genomes compared to 30% (18/59) of those infected with only the full-length virus (Fig 2B). The potential association between virological relapse and the presence of HCV defective genomes is characterized by a strong odds ratio (OR 3.35) but displayed no statistical significance by univariable analysis (Chi-test Pval 0.034, Fisher’s test Pval 0.069). This association, however, was found to be significant in a multivariate analysis that included the effect of patient IL28B genotype (see below).

### IL28B genotype profoundly influences on-treatment viral kinetics and SVR

IL28B genotype has recently emerged as a strong predictor for viral clearance in HCV1 patients undergoing PEG-IFNα/RBV therapy. Therefore, to better elucidate a possible role of HCV defective variants in influencing treatment outcome, it was essential to also take into account the well-established effect of IL28B genotype. For this reason, rs12979860 SNP genotype was determined in all the subjects of the study cohort. IL28B (rs12979860) genotype frequencies in HCV1 population were 42.4% CC, 28% CT and 29.6% TT; therefore, 57.6% of the subjects were carriers of the “non-responder” T allele. The relationships between IL28B genotype and clinical characteristics and viral kinetics are listed in Table 1. IL28B unfavorable genotype resulted associated with lower alanine transaminase (ALT) and higher gamma-glutamyl transpeptidase (GGT) pre-therapy levels (Wilcoxon test pval 0.0133 and 0.0316 respectively). Moreover, a weak association (Fisher’s test Pval 0.059) was noticeable between IL28B unfavorable genotype and the lower inflammation grading scores (not shown). As expected, IL28B unfavorable genotype strongly correlated with poorer initial response to antiviral therapy (RVR and cEVR rates, Fig 2C and 2D) as well as with treatment failure (Fisher’s test Pval 3.71e^-7^).

### The association between HCV defective particles and higher histological grading is independent from IL28B genotype and viremia

In order to confirm the correlation between increased necroinflammatory activity and the presence of HCV defective particles by also taking into account the possible role of IL28B genotype and other confounding factors, a stepwise multivariable logistic regression model was exploited. The stepwise algorithm, considering high necroinflammatory activity (grading ≥ 9) as dichotomous outcome, inserts and deletes explanatory variables in an iterative process in order to select (on the basis of the minimum AIC) the best model comprising the explanatory variables independently associated with the outcome. The variables considered as input for the selection process were: patient age, viral load, gender, BMI ≥ 25 kg/m^2^, IL28B genotype, presence of HCV defective variants. In the stepwise procedure, viral load, among other variables, was not found to be associated with grading score and therefore was not included in the model.

As shown in Table 3, the final model resulting from the stepwise procedure confirmed the role of HCV deletions (Pval 0.028, OR 2.89), along with patient age, as risk factors for higher grading index, while IL28B genotype had a modest effect (Pval 0.079, OR 0.48 for the CT/TT variants).

**Table 3:** Multivariable logistic regression for Grading, ETR and Relapse.

Factor	Multivariable Pval	OR	CI
**Outcome: Grading**			
HCV deletion	0.028	2.89	1.12–7.44
Age	0.043	1.46^a^	1.02–2.15 ^a^
IL28B CT/TT	0.079	0.48	0.23–1.02
**Outcome: ETR**			
IL28B CT/TT	3.95 e-7	0.06	0.02–0.18
Age	0.004	0.51 ^a^	0.32–0.79 ^a^
Viremia	0.035	0.96 ^b^	0.92–0.99 ^b^
GGT	0.008	0.79 ^c^	0.65–0.93 ^c^
**Outcome: Relapse**			
HCV deletion	0.049	3.73	1.01–13.77
Grading ≥ 9	0.017	4.11	1.29–13.07
IL28B CT/TT	0.021	3.90	1.23–12.33

OR: odds ratio—CI: 95% confidence interval

^a^: odds ratio and confidence intervals have been calculated considering an interval of 10 years.

^b^: odds ratio and confidence intervals have been calculated considering an interval of 10^5^ units of viremia

^c^: odds ratio and confidence intervals have been calculated considering an interval of 20 units of GGT

### IL28B genotype, increased necroinflammatory activity and defective HCV genomes are independent predictors of virological relapse

Using the same approach, we employed a multivariable logistic regression model to pinpoint the parameters associated with viral clearance at the end of treatment (ETR). Input explanatory variables for the stepwise procedure were: age, gender, viral load, BMI ≥ 25 kg/m^2^, ALT, GGT, IL28B genotype, grading ≥ 9, staging ≥ 4 (or cirrhosis), presence of HCV defective variants. In the final model selected by the procedure, summarized in Table 3, IL28B CC genotype was the variable most strongly and positively associated with ETR (Pval 3.95e-7, OR 0.06 for CT/TT variants, corresponding to an OR of 16.6 for CC genotype). Patient younger age, lower pre-treatment viral load and lower GGT levels also resulted significantly associated with ETR. The presence of defective HCV particles was not selected as covariate in the model by stepwise procedure, thus suggesting that it has no impact on ETR.

Similarly, a model was constructed to evaluate the explanatory variables predicting virological relapse in the 74 subjects who where HCV-negative at the end of treatment. As described in Table 3, the presence of deletions in HCV genome represented a risk factor for relapse with an OR of 3.73 (Pval 0.049), along with mild-to-severe histological grading (OR 4.11, Pval 0.017) and IL28B CT/TT genotypes (OR 3.90, Pval 0.021).

## Discussion

In this study, we analyzed the presence of viral defective forms in serum samples obtained from a well-characterized cohort of patients chronically infected with genotype 1 HCV. Our screening campaign, designed to detect deletion events occurring in the region of Core to NS3, revealed the presence of virus carrying large in-frame genomic deletions, located in the E1-NS2 region, in about 20% of examined sera. Our findings are in line with previously published results and highlight that all the defective genomes contain the genomic regions essential for autonomous HCV RNA replication (5′UTR and NS2-NS5-3′UTR). In addition, the Core region appears to be always preserved [3–7]. The finding that the Core region is always present in the HCV subgenomes described here could suggest that this protein is required in *cis* for efficient genome encapsidation. In a study by Steinmann et al. [17], however, the authors observed that, in cell culture, subgenomic JFH1 replicons lacking the Core to NS2 coding region could be efficiently encapsidated into infectious virus-like particles. These data suggest that Core can also be effectively provided in *trans*. This apparent discrepancy could be due to different encapsidation requirements *in vivo* compared to the cell culture system. Alternatively, the Core protein, in addition to encapsidating the viral genome, could have additional important, yet unknown functions that are more advantageously carried out in *cis*. Future research is required to address the exact nature of this phenomenon.

To our knowledge, this is the first systematic analysis carried out in a large panel of samples from well-characterized patients, and it permits to infer that the emergence of HCV deletion mutants as a circulating species, far from being a sporadic phenomenon, occurs in a relevant portion of chronically infected subjects. Furthermore, quantitative analysis carried out in a subset of patients revealed that deleted genomes are a relevant fraction of total circulating HCV.

Defective interfering viral forms have been reported for a variety of viruses and their clinical impact is variable [18]. As consequence of DI virus insurgence, a reduction in the titres of the parental virus [19] and attenuation of disease severity [20] have been reported; on the other hand, it has been proposed that DI viruses may play a role in the establishment and maintenance of persistent infection, as described for Murray Valley Encephalitis Virus and Japanese Encephalitis Virus [12,21]. Moreover, a crucial pathogenic role has been established for some DI viruses belonging to the *Flaviviridae* family. In the case of BVDV, a highly cytopathogenic viral isolate was shown to contain a DI virus lacking the genes encoding the structural proteins [11]. In fact, the pathogenesis of lethal mucosa disease in animals persistently infected with non-cytopathogenic BVDV has been ascribed to the insurgence of cytopathogenic variants containing genomic rearrangements, among which internal deletions [22]. Similarly, cytopathogenicity of CSFV was demonstrated to be associated with a DI variant lacking the structural protein-coding region [23].

Regarding HCV, the ability of defective interfering viruses to influence the clinical course of the disease is largely unexplored. Focusing on the clinical characteristics of our cohort, we observed that the presence of HCV defective variants correlates with higher viral load, older patient age and higher degree of hepatic inflammation. Since defective viruses are believed to generate from the *wt* genome through polymerase errors, the probability of their insurgence increases proportionally with the number of replication events. This may be at the basis of the association with patient older age (which could be considered as a proxy of the duration of infection) and higher viremia. From an etiopathological point of view, the association with necroinflammatory grading raises the possibility that HCV deletion mutants exacerbate liver inflammation. Given their high replication fitness and inability to autonomously exit from the cell, deleted variants are likely more prone to lead to the accumulation of pro-inflammatory viral proteins. Since the presence of an intact Core is one of the hallmarks of defective HCV genomes, it is conceivable that cells infected with subgenomic HCV will accumulate very high level of intracellular Core protein that, in contrast to the case of cells infected with the *wt* virus, will not be counterbalanced by the exit of mature viral particles. Overexpression of intracellular Core has been reported to induce the production for free radicals, oxidative stress and apoptotic cell death [24]. Moreover, Core protein has been indicated to be involved in liver steatosis, fibrosis and carcinogenesis in various mouse model systems [25].

Altered expression of other viral proteins may also negatively impact the hepatocyte function. In particular, the fusion protein produced by the deletion event may be able to exacerbate the pathological changes in the cell. Induction of ER stress has been reported in both *in vitro* models of HCV and biopsies of HCV patients [26]; since fusion proteins are probably more prone to be unfolded or misfolded, it could be speculated that ER stress is intensified in patients carriers of defective variants. A growing body of evidence in fact suggests that ER stress and the inflammatory response are strictly interconnected [27].

We did not observe a correlation between the presence of defective interfering forms and the stage of liver fibrosis. However, it should be considered that, for this cohort, the lack of knowledge of the time of infection and time of insurgence of the defective variants prevented us to correctly estimate the fibrosis progression rate and thus properly assess the impact of subgenomic HCV RNAs.

We then evaluated the viral kinetics during “classical” PEG-IFNα/RBV therapy treatment in genotype 1 HCV patients carrying the defective forms compared to those in which only the full-length form was detected. No difference was found in RVR, cEVR and ETR rates, thus indicating that defective particles do not seem to play a key role in determining viral load decline during treatment. This result was confirmed also by logistic regression analysis, in which IL28B genotype, patient age, pre-treatment viremia and GGT levels, but not the presence of defective HCV, were identified as independent predictors of ETR. Interestingly, SVR rates tended to be lower for patients with defective genomes (24% compared to 38.3%), but the difference was not found to be statistically significant. Refining the analysis on the ETR-positive population, however, we observed that defective particle carriers displayed a higher probability to experience virological relapse during the follow-up period. This result is particularly interesting, since *in vitro* experiments suggest that defective viruses belonging to the *Flaviviridae* family may play a role in the establishment and maintenance of chronic infections [12,21]. A possible role in viral persistence has been suggested also for deleted variants of hepatitis B virus (HBV). Deletions in the surface antigen regions S1 and S2 of HBV genome have been reported in sera and liver of HBV patients [28,29]. For HBV, since pre-S region contains several epitopes for T or B cells [30], the emergence of deleted variants may result in an impairment of viral clearance and therefore potentially be a mechanism of evasion of immune surveillance. Notably, HCV particles carrying defective genomes have the potential to function as”immunological decoys” capable of diverting the immune responses away from the wild type virus. This mechanism could in turn favour viral persistence and impair viral clearance.

Multivariable analysis confirmed the potential role of subgenomic HCV as independent risk factor for relapse, with an effect magnitude (OR 3.73) comparable to that of IL28B genotype (OR 3.90). Although this might suggest contributions of both the IL28B genotype and HCV defective forms in determining the pattern of response to PEG-IFNα/RBV treatment, we acknowledge that external, independent validation of our data would be necessary to confirm our findings.

Our study indicated that, in pre-treatment sera of chronic genotype 1 hepatitis C patients, HCV particles with large in frame-deletions in E1-NS2 region are detected in about 20% of the subjects and correlate with older patient age, higher viral load and with a higher degree of liver necro-inflammation measured by the Ishak score. Regarding the response to therapy, although it did not significantly impact the SVR rate, the presence of defective HCV genomes was more frequently observed in subject experiencing a viral relapse after “classical” PEG-IFNα/RBV therapy.

While the observation of the potential association with virological relapse to PEG-IFNα/RBV could represent a biologically interesting finding, it is not likely to have any practical impact in a time where relatively short therapeutic course of IFN-free, all oral combinations of direct-acting antivirals (DAA) cure nearly 100% of patients, at least for genotype 1 [31]. It is important to bear in mind, however, the recent unexpected data on decreased effectiveness of even the most advanced DAA combinations on genotype 3 infections, which represents about 10–15% of the world HCV reservoir and is associated with severe liver steatosis, accelerated fibrosis progression rate and increased oncogenesis [32].

In conclusion, our study provides new important insights in the neglected field of HCV defective variants, giving detailed information about their structure, frequency and impact on clinical characteristics. It will be very important for future studies to address the occurrence of HCV with defective genomes in other viral genotypes—especially genotype 3—and the potential association with liver diseases severity as well as response to DAA therapy.

## References

[1] Perz JF, Armstrong GL, Farrington LA, Hutin YJ, Bell BP. The contributions of hepatitis B virus and hepatitis C virus infections to cirrhosis and primary liver cancer worldwide. J Hepatol. 2006;45(4):529–38. 16879891 10.1016/j.jhep.2006.05.013

[2] Smith DB, Pathirana S, Davidson F, Lawlor E, Power J, Yap PL, et al The origin of hepatitis C virus genotypes. J Gen Virol. 1997;78 (Pt 2):321–8. 9018053 10.1099/0022-1317-78-2-321

[3] Yagi S, Mori K, Tanaka E, Matsumoto A, Sunaga F, Kiyosawa K, et al Identification of novel HCV subgenome replicating persistently in chronic active hepatitis C patients. J Med Virol. 2005;77(3):399–413. 16173026 10.1002/jmv.20469

[4] Iwai A, Marusawa H, Takada Y, Egawa H, Ikeda K, Nabeshima M, et al Identification of novel defective HCV clones in liver transplant recipients with recurrent HCV infection. J Viral Hepat. 2006;13(8):523–31. 16901282 10.1111/j.1365-2893.2006.00760.x

[5] Bernardin F, Stramer SL, Rehermann B, Page-Shafer K, Cooper S, Bangsberg DR, et al High levels of subgenomic HCV plasma RNA in immunosilent infections. Virology. 2007;365(2):446–56. 17493654 10.1016/j.virol.2007.04.003 PMC2001282

[6] Noppornpanth S, Smits SL, Lien TX, Poovorawan Y, Osterhaus AD, Haagmans BL. Characterization of hepatitis C virus deletion mutants circulating in chronically infected patients. J Virol. 2007;81(22):12496–503. 17728237 10.1128/JVI.01059-07 PMC2168980

[7] Sugiyama K, Suzuki K, Nakazawa T, Funami K, Hishiki T, Ogawa K, et al Genetic analysis of hepatitis C virus with defective genome and its infectivity in vitro. J Virol. 2009;83(13):6922–8. 10.1128/JVI.02674-08 19369330 PMC2698526

[8] Ohtsuru S, Ueda Y, Marusawa H, Inuzuka T, Nishijima N, Nasu A, et al Dynamics of defective hepatitis C virus clones in reinfected liver grafts in liver transplant recipients: ultradeep sequencing analysis. Journal of clinical microbiology. 2013;51(11):3645–52. 10.1128/JCM.00676-13 23985907 PMC3889746

[9] Pacini L, Graziani R, Bartholomew L, De Francesco R, Paonessa G. Naturally occurring hepatitis C virus subgenomic deletion mutants replicate efficiently in Huh-7 cells and are trans-packaged in vitro to generate infectious defective particles. J Virol. 2009;83(18):9079–93. 10.1128/JVI.00308-09 19587042 PMC2738267

[10] Aoki H, Ishikawa K, Sakoda Y, Sekiguchi H, Kodama M, Suzuki S, et al Characterization of classical swine fever virus associated with defective interfering particles containing a cytopathogenic subgenomic RNA isolated from wild boar. J Vet Med Sci. 2001;63(7):751–8. 11503902 10.1292/jvms.63.751

[11] Kupfermann H, Thiel HJ, Dubovi EJ, Meyers G. Bovine viral diarrhea virus: characterization of a cytopathogenic defective interfering particle with two internal deletions. J Virol. 1996;70(11):8175–81. 8892949 10.1128/jvi.70.11.8175-8181.1996 PMC190898

[12] Lancaster MU, Hodgetts SI, Mackenzie JS, Urosevic N. Characterization of defective viral RNA produced during persistent infection of Vero cells with Murray Valley encephalitis virus. J Virol. 1998;72(3):2474–82. 9499109 10.1128/jvi.72.3.2474-2482.1998 PMC109548

[13] Aghemo A, Rumi MG, Monico S, Prati GM, D'Ambrosio R, Donato MF, et al The pattern of pegylated interferon-alpha2b and ribavirin treatment failure in cirrhotic patients depends on hepatitis C virus genotype. Antiviral therapy. 2009;14(4):577–84. 19578243

[14] Bozdogan H. Model Selection and Akaike’s Information Criterion (AIC): The General Theory and Its Analytical Extensions'. Psychometrika. 1987;52:345–70.

[15] R Development Core Team. R: A Language and Environment for Statistical Computing. Vienna, Austria: the R Foundation for Statistical Computing. ISBN: 3-900051-07-0. Available: http://www.R-project.org/.

[16] Venables WN, Ripley BD. Modern Applied Statistics with S. 4th ed New York: Springer; 2002.

[17] Steinmann E, Brohm C, Kallis S, Bartenschlager R, Pietschmann T. Efficient trans-encapsidation of hepatitis C virus RNAs into infectious virus-like particles. J Virol. 2008;82(14):7034–46. 10.1128/JVI.00118-08 18480457 PMC2446957

[18] Marriott AC, Dimmock NJ. Defective interfering viruses and their potential as antiviral agents. Rev Med Virol. 2010;20(1):51–62. 10.1002/rmv.641 20041441

[19] de la Torre JC, Holland JJ. RNA virus quasispecies populations can suppress vastly superior mutant progeny. J Virol. 1990;64(12):6278–81. 2173792 10.1128/jvi.64.12.6278-6281.1990 PMC248805

[20] Aaskov J, Buzacott K, Thu HM, Lowry K, Holmes EC. Long-term transmission of defective RNA viruses in humans and Aedes mosquitoes. Science. 2006;311(5758):236–8. 16410525 10.1126/science.1115030

[21] Yoon SW, Lee SY, Won SY, Park SH, Park SY, Jeong YS. Characterization of homologous defective interfering RNA during persistent infection of Vero cells with Japanese encephalitis virus. Mol Cells. 2006;21(1):112–20. 16511353

[22] Fricke J, Gunn M, Meyers G. A family of closely related bovine viral diarrhea virus recombinants identified in an animal suffering from mucosal disease: new insights into the development of a lethal disease in cattle. Virology. 2001;291(1):77–90. 11878878 10.1006/viro.2001.1170

[23] Meyers G, Thiel HJ. Cytopathogenicity of classical swine fever virus caused by defective interfering particles. J Virol. 1995;69(6):3683–9. 7745717 10.1128/jvi.69.6.3683-3689.1995 PMC189084

[24] Farinati F, Cardin R, Bortolami M, Burra P, Russo FP, Rugge M, et al Hepatitis C virus: from oxygen free radicals to hepatocellular carcinoma. J Viral Hepat. 2007;14(12):821–9. 18070284 10.1111/j.1365-2893.2007.00878.x

[25] Fimia GM, Tripodi M, Alonzi T. Transgenic models for Hepatitis C virus pathogenesis. Cell Death Differ. 2003;10 Suppl 1:S16–8. 12655340 10.1038/sj.cdd.4401114

[26] Asselah T, Bieche I, Mansouri A, Laurendeau I, Cazals-Hatem D, Feldmann G, et al In vivo hepatic endoplasmic reticulum stress in patients with chronic hepatitis C. J Pathol. 2010;221(3):264–74. 10.1002/path.2703 20527020

[27] Zhang K. Integration of ER stress, oxidative stress and the inflammatory response in health and disease. Int J Clin Exp Med. 2010;3(1):33–40. 20369038 PMC2848304

[28] Fernholz D, Galle PR, Stemler M, Brunetto M, Bonino F, Will H. Infectious hepatitis B virus variant defective in pre-S2 protein expression in a chronic carrier. Virology. 1993;194(1):137–48. 8480417 10.1006/viro.1993.1243

[29] Melegari M, Bruno S, Wands JR. Properties of hepatitis B virus pre-S1 deletion mutants. Virology. 1994;199(2):292–300. 8122362 10.1006/viro.1994.1127

[30] Chisari FV, Ferrari C. Hepatitis B virus immunopathogenesis. Annu Rev Immunol. 1995;13:29–60. 7612225 10.1146/annurev.iy.13.040195.000333

[31] Aghemo A, De Francesco R. New horizons in hepatitis C antiviral therapy with direct-acting antivirals. Hepatology. 2013;58(1):428–38. 10.1002/hep.26371 23467911

[32] Goossens N, Negro F. Is genotype 3 of the hepatitis C virus the new villain? Hepatology. 2014;59(6):2403–12. 10.1002/hep.26905 24155107

